# Relationship between religious coping and post-traumatic stress disorder and professional quality of life of nurses working at COVID-19 wards: a descriptive correlational study

**DOI:** 10.3389/fpubh.2025.1535340

**Published:** 2025-06-20

**Authors:** Zohreh Sanavi Shiri, Pouran Tavakoli, Marzieh Momennasab

**Affiliations:** School of Nursing and Midwifery, Shiraz University of Medical Sciences, Shiraz, Iran

**Keywords:** religious and spiritual coping, post-traumatic stress disorder, quality of life, nurse, COVID-19

## Abstract

**Purpose:**

Considering effects of COVID-19 pandemic on the physical and mental health and professional quality of life (PQoL) of nurses working at COVID-19 wards, it seems necessary to investigate the factors affecting adaptation and reducing adverse effects of this pandemic on nurses. The present study aims to investigate the relationship between religious coping with post-traumatic stress disorder (PTSD) and professional quality of life (PQoL) among nurses.

**Materials and methods:**

This descriptive correlational study was conducted on 368 nurses working at hospitals affiliated to Shiraz University of Medical Sciences. In this research, Mississippi scale for post-traumatic stress disorder (M-PTSD), Pargament’s brief religious coping measure (B-RCOPE) and Stamm’s professional quality of life (proQol) were used for data collection. Statistical significance was considered at *p* < 0.05.

**Results:**

The mean score of positive religious coping was 13.01 ± 5.22 (moderate) and the mean score of negative religious coping was 5.27 ± 4.57 (low). The mean PTSD score of nurses was 96.92 ± 18.17 and most of them were at the moderate level (92.9%). The scores of compassion fatigue, secondary traumatic stress, and compassion satisfaction were in the moderate range. The Spearman’s correlation test results showed a significant and negative correlation between positive religious coping and PTSD, and a significant and positive correlation between negative religious coping and PTSD (*p* = 0.000). Moreover, a significant and positive correlation was observed between compassion satisfaction and positive religious coping (*p* = 0.005), and a negative and significant correlation was found between compassion fatigue and secondary stress and positive religious coping (*p* < 0.05). The statistical test of multiple regression revealed a significant correlation between nurses’ positive religious coping and compassion satisfaction, as well as between nurses’ negative religious coping and secondary stress and PTSD (*p* < 0.05).

**Conclusion:**

Positive religious coping was correlated with reduced PTSD and improved PQoL among nurses.

## Graphical abstract

Theoretical model to determine Relationship between Religious Coping and Post-traumatic Stress Disorder and Professional Quality of Life of Nurses Working at COVID-19 Wards.
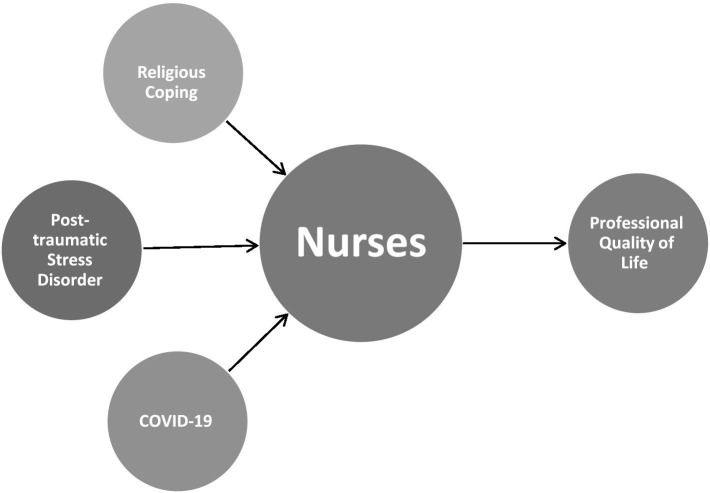

## Introduction

COVID-19 was first reported in Wuhan, China (December, 2019) and rapidly spread in whole China and, subsequently, worldwide (1, 2). The outbreak of this pandemic exposed the healthcare workers, as the frontline group, to the risk of physical and mental disorders (3). Although healthcare providers were also exposed to stress, compassion fatigue, depression, drug addiction and suicide before COVID-19 pandemic (4), initial world reports have revealed the incidence of depression, anxiety, Post-traumatic stress disorder (PTSD), suicide, compassion fatigue and sleep disorders increased among them after this pandemic (5–7). PTSD is among the psychological disorders among nurses that has been addressed by various studies during the pandemic period (8–12).

PTSD is considered a mental disorder that develops when an unfortunate and traumatic event threatens one’s life (13). Currently, PTSD is a growing concern in the nursing profession. Nurses are exposed to this disorder due to being either directly or indirectly in traumatic conditions and being responsible for taking care of patients (14).

Studies have demonstrated professional quality of life (PQoL) of healthcare providers decreased during COVID-19 pandemic (15, 16). PQoL is a multidimensional concept, consisting of two components of compassion satisfaction and compassion fatigue. The second component itself includes two components of compassion fatigue and secondary traumatic stress (17). Among all health-related professions, nurses are more prone to burnout, compassion fatigue and reduced job satisfaction due to the mental and physical stress caused by taking caring of critically ill patients (18). This issue is of particular importance because nurses’ PQoL is associated with the quality of nursing care, work productivity, burnout, job satisfaction, inappropriate performance, inappropriate violence, organizational effectiveness and organizational commitment (19–21). Currently, there is strong evidence on the inverse correlation between occupational stress and PTSD among nursing staff as well as between their perceived job satisfaction and PQoOL (22–24).

Secondary traumatic stress is considered a negative aspect in PQoL, which plays a vital role in nurses’ physical and mental health (25). During stressful events, coping strategies are stabilizing methods used to help individuals maintain psychological adaptation (26). Coping strategies could be defined as cognitive and behavioral measures used to manage stressful conditions (27). Coping strategies affect one’s feelings or thoughts to create a good quality of life and make a positive action (28). Adopting an appropriate coping strategy plays a major role in relieving PTSD symptoms (29). However, coping strategies could cause severe psychological stress, if not appropriately used (28).

Spirituality has received great attention as an important coping strategy for promoting psychological well-being among patients and healthcare workers (30). Religion is often considered an important and well-known aspect of spirituality (31). Religious coping is defined as using religious beliefs and behaviors to solve problems and prevent or relieve negative psychological consequences when faced with difficult problems and conditions (32).

Religious coping includes two positive and negative dimensions. Positive religious coping (PRC) involves ways of facing negative life events, in which one accepts the events using positive appraisals and interpretations related to God’s will. However, in other form of coping, called negative religious coping (NRC), a person establishes an avoidant and insecure relationship with God. PRC involves benefiting from a favorable connection with God or higher power during the crisis. In NRC, one blames God for their difficulties. Positive and negative religious coping is associated with higher and lower levels of psychological health, respectively (33). Studies have reported positive religious coping during infectious disease outbreaks could help individuals reduce the risk of depression (34, 35). However, some studies have not found a significant correlation between positive religious coping and lower levels of anxiety, depression and concern (36–39). The conflicting results show the necessity of conducting further studies in this field.

Considering the effect of COVID-19 pandemic on the physical and mental health and PQoL of nurses working at COVID-19 wards, it seems necessary to investigate the factors affecting adaptation and reducing adverse effects of this pandemic on nurses as their adaptation could be improved by identifying and strengthening these factors. Although the relationship between religious coping and some variables has been previously investigated separately, these variables have been less addressed during COVID-19 pandemic, which imposed a severe and chronic stress on nurses. The aim of the present study was to investigate the corellations between religious coping with PTSD and PQoL of nurses working at COVID-19 wards.

Some studies have been conducted separately on nurses’ post-traumatic stress disorder, quality of professional life, and religious adjustment and coping strategies during the COVID-19 pandemic (40–47). Studies on the relationship between religious coping and post-traumatic stress disorder have been limited and show contradictory results (38, 48, 49). However we could not find any study which investigated the multivariate relationship between all three mentioned variables. This gap in studies indicates the necessity of conducting the present study which aimed to investigate the correllations between religious coping with PTSD and PQoL of nurses working at COVID-19 wards.

## Materials and methods

### Study type and setting

In this descriptive correlational study, the nurses working at COVID-19 wards of five hospitals affiliated to Shiraz University of Medical Sciences, southern Iran, during the last 6 months.

### Sampling and sample size

Participants were selected using convenience sampling method. The sample size was determined 304 individuals based on the results of a similar study (50) using G*Power software considering the power of 80% and *α* = 0.05, which increased to 365, taking into account the withdrawal rate of 20%.

The inclusion criteria were having at least a bachelor’s degree in nursing, having at least 2 months of work experience at COVID-19 wards at the time of completing the questionnaire, not suffering from known mental illnesses such as anxiety and severe depression, not facing physical or mental traumatic events within 6 months before the study, lack of severe work or communication problems in the workplace (The last three items were based on the participants’ self-report) and being willing to participate in the study. The exclusion criterion was failure to complete the questionnaires.

### Measurements

In this study, demographic questionnaire (including age, gender, level of education, marital status, total work experience, length of time working in the COVID-19 wards, living with high-risk people, and how religious they are from their point of view), Mississippi scale for post-traumatic stress disorder (M-PTSD), Pargament’s brief religious coping measure (B-RCOPE) and Stamm’s professional quality of life (proQol) were used to collect data.

The self-report 39-item M-PTSD was developed and introduced by Norris and Raid (99). The items with a star are scored based on a 5-point Likert scale, ranging from (5) very often true to (1) never true, and items without a star are reverse-scored, and the total score ranges between 0 and 195. Scores less than 65, 65–130 and above 130 are considered mild, moderate and severe, respectively (51). The Cronbach’s alpha coefficient of this scale was reported as 0.86–0.94 (52). In the present study, the Persian version of this scale, which has been previously used in various studies in Iran and its validity and reliability have been confirmed, was employed (51–54). This scale was validated by Goodarzi (51) in Iran. The scale reliability was obtained as 0.92, 0.92, 0.91, and 0.82 based on the internal consistency, split-half method, test–retest method and peer test (PTSD inventory) (53). In the present study, Cronbach’s alpha for the M-PTSD questionnaire was determined to be 0.88.

The 14-item brief religious coping measure (B-RCOPE) was developed by Pargament (100). In this questionnaire, each of the positive and negative scales includes 7 items. Items 1–7 indicate positive religious coping and items 8–14 indicate negative religious coping. The items are scored based on a 4-point Likert scale, ranging from “never” to “always” (0–3). The minimum and maximum scores of each subscale of positive and negative religious coping are 0 and 21, respectively. The religious orientation scale (ROS) was used as a reference to assess the reliability and validity of the Persian version of this scale in a preliminary study conducted in 2001. Concurrent validity was assessed using religious coping scale. The correlation between the scores obtained from the simultaneous use of the two scales was obtained as 0.6 (101, 102). Cronbach’s alpha coefficient was calculated to evaluate the scale validity. This coefficient was obtained as 0.86 and 0.65 in the subscales of positive and negative religious coping, respectively (55). In this study, the values of Cronbach’s alpha coefficient were obtained as 0.90 and 0.83 for positive and negative religious coping, respectivelyThe self-report 30-item professional quality of life (proQol) was designed and developed by Stamm. This scale includes three dimensions of compassion satisfaction (items 3, 6, 12, 16, 18, 20, 22, 24, 27, 30), compassion fatigue (items 1, 4, 8, 15, 10, 17, 19, 21, 26, 29) and secondary traumatic stress (items 2, 5, 7, 9, 11, 13, 14, 23, 25, 28). Each dimension consists of 10 items scored based on a 5-point Likert scale (1 = never to 5 = very often). The score of each subscale is obtained from the sum of all its items, so that the score of each subscale varies between 10 and 50. Each subscale consists of 10 items. In all the three subscales, the score of 22 or less indicates low level of compassion satisfaction, compassion fatigue and secondary traumatic stress, the score of 23–41 indicates moderate level of compassion satisfaction, compassion fatigue and secondary traumatic stress, and the score of 42 and higher indicates high level of compassion satisfaction, compassion fatigue and secondary traumatic stress (56). In the present study, the Persian version of this scale, which has been previously used in various studies in Iran and its validity and reliability have been confirmed, was employed. The validity and reliability of the whole scale was confirmed in the study by Yadollahi et al. and its Cronbach’s alpha was reported as 0.70 (57). In other studies, the alpha coefficient was reported as 0.73 and 0.75 (58, 59). In the present study, the Cronbach coefficient was estimated as 0.88, 0.74, 0.84, and 0.65 for compassion satisfaction, compassion fatigue, secondary traumatic stress, and proQol total use score, respectively.

### Data collection

The samples were first selected using the convenience sampling method considering the inclusion criteria. After explaining the research objectives and methodology, the participants signed the informed consent form. The questionnaires were given and collected by researcher (first author) and her assistant and collected after completing. Due to the multiplicity of questionnaires and the large number of questions and in order to maintain the cooperation of the samples, the questionnaires were collected 2 days after distribution. In total, 375 questionnaires were completed, 7 of which were excluded due to high missing values, and the data of 368 nurses were analyzed.

### Statistical analysis

The data were analyzed using SPSS 26.0. Given that the data were not normally distributed, non-parametric Mann–Whitney and Kruskal-Wallis tests were used. Pearson’s correlation coefficient and its non-parametric equivalent, i.e., Spearman’s correlation, as well as multiple regression were employed to investigate the correlation between the quantitative variables. *p*-value < 0.05 was considered statistically significant.

### Ethical considerations

This study was approved by Research Ethics Committee of Shiraz University of Medical Sciences (No: IR.SUMS.NUMING.REC.1401.30). All the participants signed the informed consent form after receiving information about research objectives and methodology. The participants were assured that their information would be kept confidential and anonymous. It was emphasized that it would not have any impact on their professional status if they do not participate or withdrawal from the study.

## Results

The mean age of the nurses participating in the study was 31.43 ± 6.45 years old, ranging from 22 to 55 years old. The mean work experience of nurses was 8.21 ± 6.44, ranging from 6 months to 28 years, and the mean work experience at the COVID-19 ward was 16.11 ± 10.91, ranging from 2 to 36 months. Most of the nurses were female (77.40%) and married (54%) and had a bachelor’s degree (90%). The majority of the nurses were working at non-intensive units (61%) and did not live with older adult people (61.5%) or those with chronic diseases (85.50%). Most of them considered themselves to be moderately religious (54%) (Table 1).

**Table 1:** Mean score of religious coping considering demographic characteristics of nurses working at COVID-19 ward.

	Classification	Frequency (%)	Mean	Test statistic	*p*-value
Gender	Female	285 (77.44%)	177.81	−1.73	0.083^*^
	Male	83 (22.55%)	200.63		
Educational level	Bachelor’s degree	318 (89.83%)	183.37	2.00	0.001^*^
	Master’s degree or Ph.D.	36 (10.16%)	206.41		
Marital status	Single	169 (46.42%)	165.95	−0.64	0.518^*^
	Married	195 (53.57%)	179.19		
Living with parents or older adult people	Yes	139 (38.61%)	188.42	−1.14	0.251^*^
	No	221 (61.38%)	175.52		
Living with high-risk people or those with special diseases	Yes	52 (4.68%)	171.33	−0.47	0.637^*^
	No	302 (85.31%)	178.56		
Ward type	Intensive care unit	131 (38.87%)	158.04	−1.64	0.099^*^
	Non-intensive care unit	206 (61.12%)	175.97		
Self-report religiosity	High	27 (7.50%)	256.13	2.00	0.000^**^
	Moderate	246 (68.33%)	193.83		
	Low	87 (24.16%)	119.33		
Having a history of chronic diseases	Yes	35 (9.91%)	185.13	−0.49	0.619^*^
	No	318 (90.08%)	176.11		

^*^Mann–Whitney test, ^**^Kruskal-Wallis.

The mean score of positive religious comping was 13.01 ± 5.22, which was at the moderate level. Also, the mean score of negative religious coping was 4.57 ± 5.27, which was at the low level. A significant correlation was observed between religious coping and educational level (*p* = 0.03) and rself-report eligiosity (*p* = 0.000).

**Figure 1:**
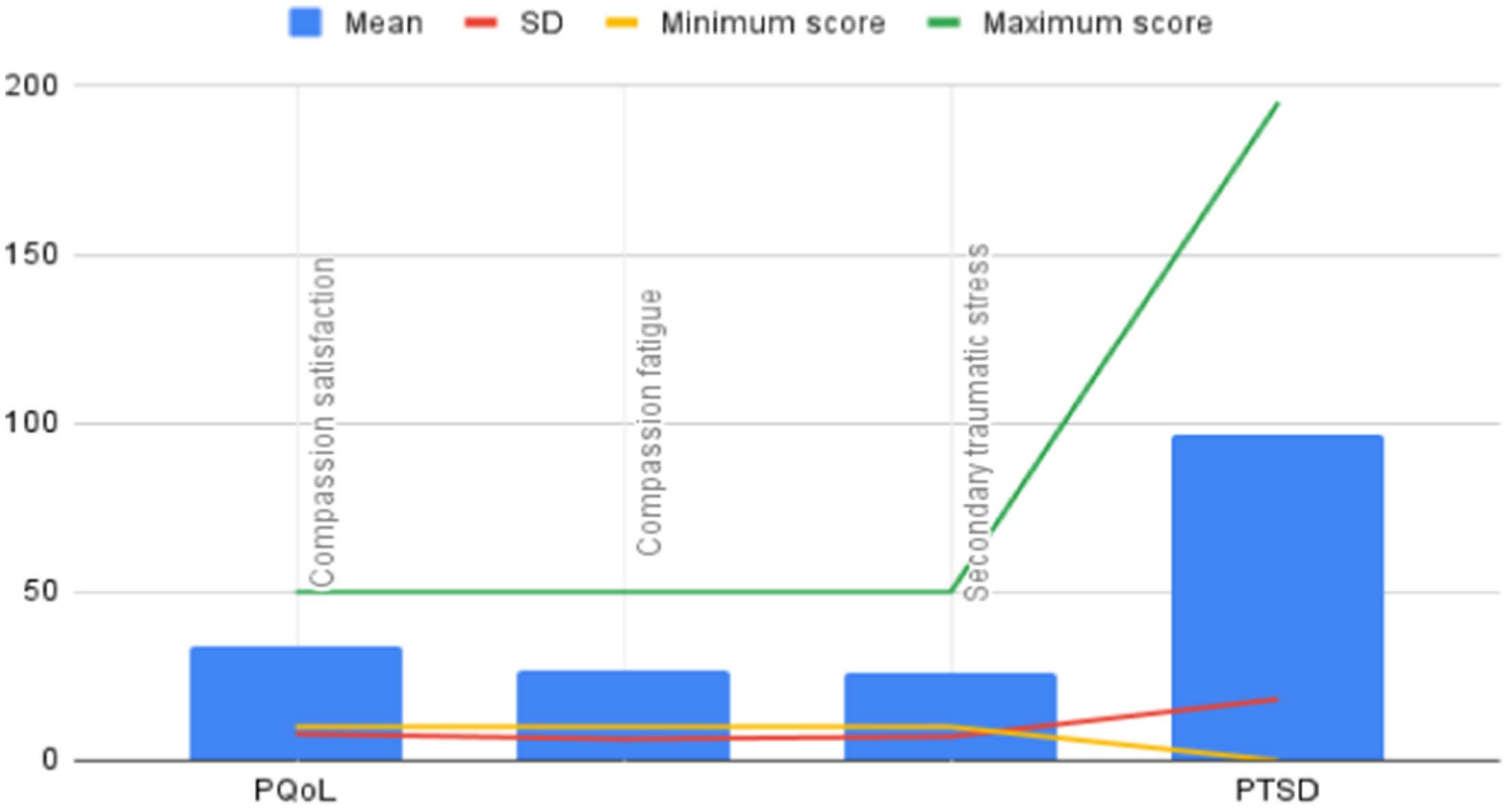
Mean and standard deviation of PQoL and PTSD scores of nurses working at COVID-19 ward.

Figure 1 presents the mean and standard deviation of PQoL and PTSD scores.

The Spearman’s correlation test results showed a significant, negative correlation between positive religious coping and PTSD and a significant positive correlation between negative religious coping and PTSD (*p* < 0.05). PTSD had a stronger correlation with negative religious coping (*r* = 0.485) (Table 2). The Spearman’s correlation test results showed a positive significant correlation between compassion satisfaction and positive religious coping (*p* = 0.005) and a significant, negative correlation between compassion fatigue and secondary stress and positive religious coping (*p* < 0.05). There was a significant and adverse correlation between negative religious coping and compassion satisfaction, and a positive and significant correlation between compassion fatigue and secondary stress (*p* < 0.05) (Table 2).

**Table 2:** Correlation between mean scores of nurses’ religious coping and PTSD.

	Total score	Intrusive memories	Difficulty in interpersonal communication	Inability to control emotions	Depression
Positive religious coping	*p* = 0.000	*p* = 0.000	*p* = −0.017	*p* = 0.000	*p* = 0.000
	*r* = −0.225	*r* = −0.185	*r* = −0.125	*r* = −0.191	*r* = −0.208
Negative religious coping	*p* = 0.000	*p* = 0.000	*p* = 0.000	*p* = 0.000	*p* = 0.000
	*r* = 0.485	*r* = 0.466	*r* = 0.256	*r* = 0.312	*r* = 0.433

The statistical test of multiple regression revealed a significant correlation between positive religious coping and compassion satisfaction among nurses (*p* < 0.05). If considering PTSD constant and adding one point to the positive religious coping score, 0.375 point will be added to the compassion satisfaction score. No significant correlation was found between positive religious coping and PTSD (*p* = 0.289). Also, no significant correlation was observed between compassion fatigue, secondary stress, PTSD and positive religious coping (*p* > 0.05) (Table 3).

**Table 3:** Correlation between mean scores of nurses’ religious coping and PQoL dimensions.

	Total score	Compassion satisfaction	Compassion fatigue	Secondary stress
Positive religious coping	*p* = 0.770	*p* = 0.000	*p* = 0.000	*p* = 0.034
	*r* = 0.015	*r* = 0.395	*r* = −0.277	*r* = −0.111
Negative religious coping	*p* = 0.000	*p* = 0.000	*p* = 0.000	*p* = 0.000
	*r* = 0.350	*r* = − 0.290	*r* = 0.420	*r* = 0.462

The statistical test of multiple regression revealed a significant correlation between negative religious coping and secondary stress and PTSD among nurses (*p* = 0.000). If considering PTSD constant and adding one point to the negative religious coping score, 0.294 point will be added to the secondary stress score. If considering secondary stress constant and adding one point to the negative religious coping score, 0.247 point will be added to the PTSD score. However, no significant correlation was found between compassion fatigue, compassion satisfaction and negative religious coping (*p* > 0.05) (Table 4).

**Table 4:** Multivariate correlation between compassion satisfaction, compassion fatigue, secondary stress and PTSD with positive and negative religious coping.

Religious coping	PTSD	Non-standard coefficient B	Standard coefficient B	*R*-squared	*R*	Sig.
Positive religious coping	Compassion satisfaction	0.245	0.375	0.165	0.406	*p* = 0.000
	Compassion fatigue	−0.012	−0.015			*p* = 0.871
	Secondary stress	0.047	0.065			*p* = 0.350
	PTSD	−0.021	−0.075			*p* = 0.289
Negative religious coping	Compassion satisfaction	−0.019	−0.320	0.305	0.552	*p* = 0.646
	Compassion fatigue	0.047	0.066			*p* = 0.438
	Secondary stress	0.190	0.294			*p* = 0.000
	PTSD	0.062	0.247			*p* = 0.000

## Discussion and conclusion

Results of the present study showed the mean scores of positive and negative religious coping were at the moderate and low levels, respectively, and positive religious coping had a higher mean score than negative religious coping. In studies by Ken Chow et al. and Francis et al. on healthcare workers working in a hospital during COVID-19 pandemic in Malaysia, the mean score of positive and negative religious coping was greater than the study present. However, the mean positive religious coping score was higher than the negative religious coping score, which was in line with the present study (41, 60). In some other studies conducted in Asian countries, positive religious coping was used more than negative religious coping (61, 62). It seems that healthcare workers tend to rely more on God or supreme existence under critical conditions (63). Not surprisingly, participants with greater self-report religiosity were significantly more likely to use religious coping. Religious coping was, in turn, associated with greater mental health, in line with previous research (64–67). The results showed significant correlation between religious coping and educational level. Although in one study there was no correlation between education level and religious coping (68), in some another studies educational level was directly correlated with spiritual experiences and religious coping (69, 70). These difference may stem from participants charactristics and cultural background.

In the present study, more than 90% of the nurses working at COVID-19 wards had moderate PTSD. Various studies have confirmed the increased incidence of PTSD at moderate and severe levels among healthcare providers and nurses working at COVID-19 wards (71–73).

In studies by Almeida and Mirab Zadeh Ardekani, the mean PTSD score has been at the severe level among about half of the participants, and the risk of suffering from PTSD in Almeida’s study was higher than that of the present study. It seems that these investigations were carried out during the COVID-19 pandemic in the absence of sufficient protective facilities when there was difficulty in controlling the disease, which brought more stress (8, 74).

The scores of all the three dimensions of PQoL scale, including compassion satisfaction, compassion fatigue and secondary stress, were at the moderate level, and compassion satisfaction had a higher mean score. Various studies conducted during the COVID-19 pandemic in the United States, as well as studies by Carla Serrão and Cuartero-Castañer in Portugal and Ecuador have shown the moderate level of compassion fatigue and secondary traumatic stress and moderate to high levels of compassion satisfaction (75–77). However, another research indicated most of the healthcare professionals had high levels of compassion fatigue and moderate to high levels of burnout and compassion satisfaction (78). Trumello et al. found that those working in areas with higher likelihood of disease transmission reported a higher level of compassion fatigue and a lower level of compassion satisfaction (79).

The conflicting results could be due to the point that these studies were mainly conducted in the initial peaks of COVID-19, while more adaptation was achieved over time using coping strategies. On the other hand, the present study was conducted after vaccination against COVID-19, which could affect the stress level and, as a result, nurses’ PQoL (80).

This study showed PTSD was significantly and inversely correlated with positive religious coping, and there was a relatively strong positive correlation between PTSD and negative religious coping. Moreover, there was a negative and significant correlation between intrusive memories, difficulty in interpersonal communication, inability to control emotions and positive religious coping. Also, a significant and positive correlation was observed between intrusive memories, difficulty in interpersonal communication, inability to control emotions, loss and depression and negative religious coping. In general, PTSD had a stronger correlation with negative religious coping, and the total religious coping score had the maximum correlation with intrusive memories and minimum correlation with inability to control emotions.

Studies by Reiner Fuentes-Ferrada and Tatum Feiler demonstrated negative religious coping was among the risk factors for PTSD among students, which was in line with results of our study (35, 81). Investigations have revealed respondents who used positive religious coping showed less PTSD symptoms than those who used negative religious coping (82–84).

There was a significant, positive correlation between compassion satisfaction and positive religious coping and a negative and significant correlation between compassion fatigue and secondary stress and positive religious coping. A significant and negative correlation was found between negative religious coping and compassion satisfaction. However, a significant and positive correlation was observed between negative religious coping and compassion fatigue and secondary stress.

Moreover, there is a significant correlation between nurses’ PQoL and religious coping. Nurses’ PQoL could be improved by promoting the quality of religious coping because religious people with high spirituality experience higher compassion satisfaction and lower compassion fatigue and burnout (85–87). It seems that people see themselves connected to a source of energy by taking advantage of religious beliefs and deal with burnout and fatigue with more tolerance using better coping strategies (56). This issue is more tangible in Iran, which has a religious background (88).

In this study, all PTSD dimensions were significantly and negatively correlated with compassion satisfaction, and all PTSD dimensions were significantly and positively correlated with compassion fatigue. Also, all PTSD dimensions were significantly and positively correlated with secondary stress. The strongest correlation was observed between intrusive memories and compassion fatigue. Some other studies have confirmed these findings (89–91).

Results of this study indicated positive religious coping had a greater impact on the compassion satisfaction than PTSD. Also, negative religious coping affected the secondary stress and PTSD, and negative religious coping had a greater effect on the secondary stress. Studies have revealed improving the quality of positive religious coping could increase nurses’ QOL (85) and positive religious coping strategies have been associated with promoting well-being and personal growth (92). Religiosity and spirituality are resources that provide a coping strategy for mental disorders, especially under stressful conditions (93). Various studies have shown negative religious coping is associated with increased psychological complications and disorders, secondary stress and post-traumatic stress (60, 82, 83, 94). Also some recent works on religious coping and mental health wellbeing among healthcare workers, found that healthcare workers employed more positive than negative coping strategies when dealing with their struggles (60, 61, 95). It seems that the positive religious coping is associated with better physical and mental health outcomes (96). Research has shown benefits of positive religious coping on mental health and opposite results of negative religious coping in different populations (97, 98).

## Conclusion

The results showed religious coping could affect nurses’ PQoL and PTSD working at COVID-19 wards. Positive religious coping was correlated negatively with PTSD and positively with PQoL. PTSD had a stronger correlation with negative religious coping.

The effect of COVID-19 pandemic on nurses’ physical and mental health and PQOL working at COVID-19 wards, which may continue for years, demonstrates the necessity of reducing the negative effects of this pandemic on nurses. Training and using different strategies to strengthen effective coping, especially religious coping, could be considered to better adapt nurses to the effects of the pandemic crisis. Holding educational workshops and in-service classes could affect nurses’ performance and viewpoints in this regard. Moreover, specialized psychological and religious counseling is recommended for nurses with high stress levels and negative religious coping users.

Although multicenter designing is the strength of this study which enhances the generalizability of the results, conducting it in a specific cultural background may limit the generalizability of the findings to other regions or healthcare settings. Another limitation of this study was that high workload and fatigue of nurses working in the hospital may have affected their responses, which could not be controlled. Furthermore, the data collection through self-report measures, may have affected accuracy of the data.

## Data Availability

The raw data supporting the conclusions of this article will be made available by the authors without undue reservation.
